# Hospitalizations for congenital syphilis in children under one year old in the state of Pará, Brazilian Amazon: ecological study

**DOI:** 10.1186/s12887-023-04409-z

**Published:** 2023-11-20

**Authors:** Débora Talitha Neri, Amanda Loyse da Costa Miranda, Eliã Pinheiro Botelho, Rubenilson Caldas Valois, Geyse Aline Rodrigues Dias, Andressa Tavares Parente, Eliete da Cunha Araújo, Glenda Roberta Oliveira Naiff Ferreira

**Affiliations:** 1 Programa de Pós Graduação em Enfermagem, Federal University of Para, Rua Augusto Correa, 01 – Setor Saúde. Guamá, 66075-110 Belém, PA Brazil; 2 State University of Pará, Belém, PA Brazil

**Keywords:** Congenital Syphilis, Hospitalization, Spatial analysis, Access to Health Services, Geographic mapping

## Abstract

**Background:**

The high incidence of congenital syphilis shows flaws in the resolution of primary health care, being a predictor of greater use of hospital services, whose regional differences in access to health actions and services may be reflected in health inequalities.

**Objective:**

to investigate hospitalizations due to congenital syphilis in children under one year of age, in the state of Pará, Brazilian Amazon.

**Methods:**

an ecological study was carried out, using hospitalization, lethality and mortality rates related to congenital syphilis in children under one year of age. Temporal analysis and mapping of hospitalization flows were carried out using Joinpoint®, version 4.7.0.0, Terraview 4.2.2, Tabwin 4.1.5.

**Results:**

A total of 6,487 hospitalizations were recorded. For the ten years of the study period (2009 to 2018), the lethality rate showed a decreasing trend of – 13.5% (*p* = 0.01). The crude hospitalization rate showed an increasing trend of 12.8% (*p* < 0.000. The regression analysis demonstrated that there was a change point in the trend with a significant growth of 12.8% until 2016 (*p* = 0.0006). In the mortality rate the trend was stable (*p* = 0.56). The analysis of hospitalization care flows made it possible to identify that most hospitalizations due to congenital syphilis occurred in the municipalities of residence, but 1,378 (21.2%) had to move. Two large care gaps were highlighted in Metropolitan health regions II and III, belonging to macroregion II. The hospitalizations of residents of these regions were carried out by the assistance networks of Belém (capital) and Marituba, both of which are part of Metropolitana I. Residents of macroregions III and IV had the greatest distances traveled to access hospital care.

**Conclusions:**

The increase in the rate of hospitalizations with an increasing trend demonstrates the impact that syphilis still causes in Brazil, not being resolved even after national government interventions in primary health care, but there was a decreasing trend in the fatality rate. The results demonstrate a heterogeneous organization of health care networks in the state’s health regions and macroregions.

## Background

Congenital syphilis (CS) still persists with a high incidence in several countries. In the Americas, the distribution is heterogeneous, and only seven countries managed to achieve the elimination of vertical transmission of syphilis, with Cuba being the first country in the world to achieve the goal [1, 2]. Brazil is responsible for the majority of cases in the region [1], but the incidence of CS varies between regions and within municipalities within a state [3]. This epidemiological scenario required adherence to international strategies and the implementation of national protocols for the control and elimination of this disease, but the incidence rate remains above 0.5 cases per thousand live births, with an increasing trend even after the implementation of actions aimed at prenatal care in primary health care (PHC) [1–4].

The Brazilian Ministry of Health recommends seven or more prenatal care visits during prenatal care and the use of the reverse sequence algorithm for diagnosing syphilis for PHC services. The rapid test for the detection of antibodies against T. pallidum was decentralized to PHC services in 2012 and, consequently, the treatment [4, 5]. The greater availability of prenatal screening for the bacteria had no impact on cases of congenital CS [3].

CS is a sensitive condition for PHC, an indicator of access and quality of PHC and to hospital care, and it is known internationally as an ambulatory care sensitive condition [6, 7]. The disease is associated with a lack of resolution in the management of gestational syphilis (GS) during prenatal care in PHC, as evidenced by the high number of pregnant women who underwent prenatal care, but their children were born with CS [3]. Diagnosis in the first and third trimesters reduces the number of cases of newborns with CS [8]. Free access to tests, administration of penicillin and follow-up of pregnant women with positive results are important low-cost interventions to reduce this negative outcome [2] and which may have an impact on the need for hospitalization [6, 9].

Access to tertiary care depends on an organized Health Care Network [4], but the Brazil’s national health system, called the Unified Health System (Sistema Único de Saúde, acronym SUS, in portuguese) is marked by regional differences in the provision and qualification of care services for labor and birth [10, 11]. The North and Northeast regions of Brazil present the worst results in terms of infrastructure related to maternity, with the lowest percentages of adult and neonatal intensive care unit beds in government-funded hospitals, lower availability of medicines and essential and strategic equipment to enable maternal survival and of the newborn in emergencies and lower number of professionals specializing in the maternal and child area [10]. In these regions, deaths in newborns were associated with pilgrimage in search of health services before birth, the absence of prenatal care and a professional to assist birth, in addition to the long wait for care before birth [11].

Gaps in care are aggravated by the long distances traveled to access health services and by the geographic barriers that exist in the Amazon region. To reduce these barriers to access, in primary health care, the National Policy established two models of Family Health teams that consider the specificities of the region: the riverside family health team and the River Family Health Teams. The latter works in basic river health units, which are large mobile river units. These techno-assistance models make it possible to reduce barriers related to access difficulties for populations living in the Amazon [12, 13] Reducing inequalities in access to health actions and services with impacts on indicators is a challenge for health systems, even in countries that have implemented regionalized networks to organize and expand access [14, 15].

Considering this context, there are few specific ecological studies on CS in children under one year of age that use data from the indicator hospitalizations for sensitive conditions in PHC [9]. Most studies carried out with data from the indicator evaluate all causes of hospitalizations in children under one year of age [6, 16] or evaluate the detection rates of CS [3]. Therefore, this study is important to understand the magnitude of hospitalizations for CS in a state in the Brazilian Amazon through an ecological study. In addition, it is important to identify the effect of implementing a population-level intervention [7, 17], such as the maternal and child health care network in these hospitalizations, which was established in 2011 in Brazil [4].

Some of the actions of the maternal and child network, implemented in the SUS, are as follows: providing prenatal care at the Basic Health Unit with early recruitment of pregnant women and qualification of care; prevention and treatment of STDs/HIV/AIDS and hepatitis; support for pregnant women in traveling to prenatal consultations and to the place where the birth will take place, which will be regulated in a specific normative act; and sufficiency of obstetric and neonatal beds (intensive care unit and intermediate care unit) according to regional needs [4].

Considering this population intervention [4], it is important to know to what extent the organization of the maternal and child network called the “stork network” contributed to the reduction of hospitalizations due to CS and improved access to hospital care in Pará. Thus, this study aimed to investigate hospitalizations by SC in children under one year of age, in the state of Pará.

## Methods

### Study design

This ecological study, which analyzed hospitalizations for CS in children under one year old that occurred from 2009 to 2018, in residents of the state of Pará. The ecological study is used when large-scale data comparisons are needed to study the effect of population-level exposures on a disease condition [18].

We sought to analyze all hospitalizations for SC paid by SUS in the period from 2009 to 2018, considering the government interventions that occurred in this period that could impact these hospitalizations. Data collection took place from May to July 2019.

The study period was chosen to evaluate the effect of a population-based intervention related to maternal and child health, the so-called “stork network”. The intervention is part of the implementation of health care networks that were implemented in Brazil. The “stork network” was created in Brazil in 2011. Temporal and spatial analysis techniques were used. Temporal trend analysis was applied to identify changes in hospitalization rates for CS during the period from 2009 to 2018. To identify the flows, travel route and travel distance of newborns who required hospitalization for CS, two periods were used: 2009–2013 and 2014–2018. The first characterizes the phase before and during the implementation of the stork network in Brazil, while the second period is the phase of stork network actions already implemented.

### Setting

**Fig. 1:**
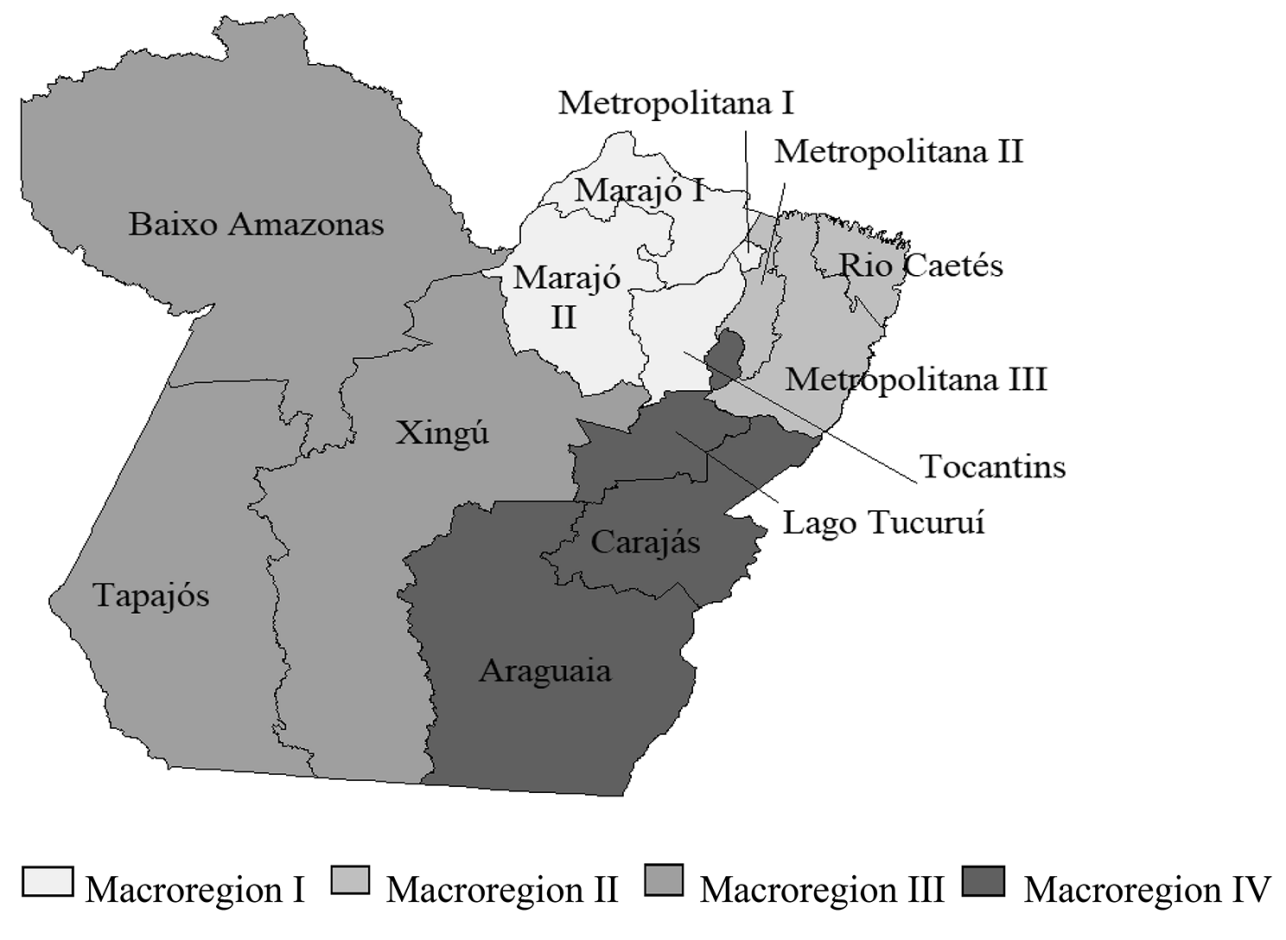
Health Regions. The map was constructed by the authors (elaborate in Tabwin program)

The state of Pará is located in the Brazilian Amazon, with a territorial extension of 1,247,955.238 square kilometers, in which 7,581,051 people live in 144 municipalities, grouped into thirteen health regions (HR): Metropolitana I, Tocantins, Marajó I, Marajó II, Metropolitana II, Metropolitana III, Caetés River, Lower Amazon, Tapajós, Xingú, Lake Tucuruí, Carajás, and Araguaia (Fig. 1). In January 2021, the state had 1,506 Family Health Teams and 50 Primary Care Teams financed by the Ministry of Health. The population covered by Family Health was 3,485, 570 people and 889 by primary care teams [19, 20].

### Participants

The analyses and results of ecological studies are applicable only at the population level, and the unit of observation is the population or community [18]. Data on hospitalizations of children under one year of age with congenital syphilis were eligible.

Inclusion criteria: records of hospitalizations for congenital syphilis of children of both sexes hospitalized during the study period in hospitals in Pará, with codes from the International Classification of Diseases: A50.0 - early CS, symptomatic; A50.1- early CS, latent; A50.2 - early CS, unspecified; A50.3 - late congenital syphilitic oculopathy, A50.4 -late congenital neurosyphilis, A50.5 - other late CS, symptomatic; A50.6 - late CS, latent; A50.7 - late CS, unspecified; A50.9- CS, Unspecified.

Exclusion criteria: hospitalization data from residents of other states in Brazil or those with incomplete records were excluded.

### Variables

The variable in this ecological study was an aggregate measure [18]. This is an attribute of people in which data on hospitalizations for congenital syphilis in children under one year of age hospitalized and residing in Pará were aggregated, according to the inclusion criteria. Data were aggregated by place of residence, place of hospitalization and year of hospitalization. The location includes municipality, health regions and macroregions.

The extracted variables or the study were clinical classification of congenital syphilis (codes A50.0, A50.1, A50.2, A50.3, A50.4, A50.5, A50.6, A50.7, A50.9), city of hospitalization and residence, death, health region, number of hospitalizations and year.

In the trend analysis, the dependent variable was the rates, and the independent variable was the years of study.

### Data sources

The data sources were hospitalizations from the Hospital Information System of the Unified Health System, extracted using the TabWin 4.1.5 program, and the number of live births from the Live Births Information System extracted by TabNet.

The purpose of the Hospital Information System is to transcribe all services arising from hospital admissions that were financed by Brazil’s single health system and, after processing, generate reports for managers that enable them to make payments to health establishments. The Live Birth Information system aims to register Live Birth declarations to support knowledge of the health situation in relation to births occurring in Brazil [21].

The Tab program for Windows and the TabNet application are research, extraction, tabulation and data crossing tools, with open and free access, which enable the general population and managers to obtain diverse information within the scope of the SUS, which is important in the management of health policies. Both allow you to quickly and safely search for data in official sources from all available Unified Health System information systems [21].

### Statistical methods

Data were stored in a Microsoft Excel spreadsheet, excluding inconsistencies after double checking (duplicity of registration, incomplete information on the place of residence and hospitalization). The description of the historical series was presented using absolute frequencies and rates. For the crude hospitalization rate, the following calculation method was adopted:

$$\frac{\text{N}\text{u}\text{m}\text{b}\text{e}\text{r} \text{o}\text{f} \text{h}\text{o}\text{s}\text{p}\text{i}\text{t}\text{a}\text{l}\text{i}\text{z}\text{a}\text{t}\text{i}\text{o}\text{n}\text{s} \text{p}\text{e}\text{r} \text{S}\text{C} \text{i}\text{n} \text{t}\text{h}\text{e} \text{p}\text{e}\text{r}\text{i}\text{o}\text{d} \left(\text{i}\text{n} \text{c}\text{h}\text{i}\text{l}\text{d}\text{r}\text{e}\text{n} \text{u}\text{n}\text{d}\text{e}\text{r} \text{o}\text{n}\text{e} \text{y}\text{e}\text{a}\text{r} \text{o}\text{l}\text{d}\right)}{\text{T}\text{o}\text{t}\text{a}\text{l} \text{n}\text{u}\text{m}\text{b}\text{e}\text{r} \text{o}\text{f} \text{l}\text{i}\text{v}\text{e} \text{b}\text{i}\text{r}\text{t}\text{h}\text{s} \left(\text{L}\text{B}\right) \text{o}\text{f} \text{m}\text{o}\text{t}\text{h}\text{e}\text{r}\text{s} \text{r}\text{e}\text{s}\text{i}\text{d}\text{i}\text{n}\text{g} \text{i}\text{n} \text{t}\text{h}\text{e} \text{p}\text{e}\text{r}\text{i}\text{o}\text{d} } \times \text{1,000}$$

The crude mortality rate used:

$$\frac{\text{N}\text{u}\text{m}\text{b}\text{e}\text{r} \text{o}\text{f} \text{d}\text{e}\text{a}\text{t}\text{h}\text{s} \text{p}\text{e}\text{r} \text{S}\text{C} \text{i}\text{n} \text{t}\text{h}\text{e} \text{p}\text{e}\text{r}\text{i}\text{o}\text{d} \left(\text{i}\text{n} \text{c}\text{h}\text{i}\text{l}\text{d}\text{r}\text{e}\text{n} \text{u}\text{n}\text{d}\text{e}\text{r} \text{o}\text{n}\text{e} \text{y}\text{e}\text{a}\text{r} \text{o}\text{l}\text{d}\right)}{\text{T}\text{o}\text{t}\text{a}\text{l} \text{n}\text{u}\text{m}\text{b}\text{e}\text{r} \text{o}\text{f} \text{l}\text{i}\text{v}\text{e} \text{b}\text{i}\text{r}\text{t}\text{h}\text{s} \left(\text{L}\text{B}\right) \text{o}\text{f} \text{m}\text{o}\text{t}\text{h}\text{e}\text{r}\text{s} \text{r}\text{e}\text{s}\text{i}\text{d}\text{i}\text{n}\text{g} \text{i}\text{n} \text{t}\text{h}\text{e} \text{p}\text{e}\text{r}\text{i}\text{o}\text{d} } \times \text{1,000}$$

Meanwhile, the lethality rate was calculated by:

$$\frac{\text{N}\text{u}\text{m}\text{b}\text{e}\text{r} \text{o}\text{f} \text{d}\text{e}\text{a}\text{t}\text{h}\text{s} \text{p}\text{e}\text{r} \text{S}\text{C} \text{i}\text{n} \text{t}\text{h}\text{e} \text{p}\text{e}\text{r}\text{i}\text{o}\text{d} \left(\text{i}\text{n} \text{c}\text{h}\text{i}\text{l}\text{d}\text{r}\text{e}\text{n} \text{u}\text{n}\text{d}\text{e}\text{r} \text{o}\text{n}\text{e} \text{y}\text{e}\text{a}\text{r} \text{o}\text{l}\text{d}\right)}{\text{T}\text{o}\text{t}\text{a}\text{l} \text{h}\text{o}\text{s}\text{p}\text{i}\text{t}\text{a}\text{l}\text{i}\text{z}\text{a}\text{t}\text{i}\text{o}\text{n}\text{s} \text{p}\text{e}\text{r} \text{S}\text{C} \text{i}\text{n} \text{t}\text{h}\text{e} \text{y}\text{e}\text{a}\text{r} \left(\text{i}\text{n} \text{c}\text{h}\text{i}\text{l}\text{d}\text{r}\text{e}\text{n} \text{u}\text{n}\text{d}\text{e}\text{r} \text{o}\text{n}\text{e} \text{y}\text{e}\text{a}\text{r} \text{o}\text{l}\text{d}\right)} \times 100$$

Trend analysis was performed based on annual percentage change estimates (annual percentage change, APC), 95% confidence interval and significance level p value < 0.05 of the rates from 2009 to 2018, which were considered increasing trends when APC positive and decreasing when APC negative had both p value < 0.05; a stationary trend was considered when p-value > 0.05. The Joinpoint® program regression model, version 4.7.0.0 (National Cancer Institute, Calverton, MD. USA), was used. The default configuration of the program was adopted to obtain the maximum number of change points. The best-fitting joinpoint regression model was accessed by the Monte Carlo permutation test, which employed 4,999 permutations. In this regression model, joinpoints are fitted in a linear regression until the joints distinguish the trend periods.

For mapping the care flows, using the Tabwin program (Version 4.15), the flow table was extracted, with analysis in the Terraview program. The origin (municipality of residence) and destination (municipality of hospitalization) were considered, to know the distance traveled for hospitalization, flows between municipalities, the assistance networks formed and the typology of flows between the assistance networks.

The flows of the hierarchical direct ascending (HDA) follow the structure of the network and go to higher level centers, being directly subordinate to these; horizontal transversal between networks (HTN) are those that occur between centers of the same level and of different networks; Ascending transversals between networks (ATN) are those that occur between different networks as well, but between centers of different levels, they always go to centers of higher level.

To analyze the flows (map), an ‘origin-destination pair’ was defined, characterized by the connection between the place of residence and the place of hospitalization, these being two points joined by an indivisible line. Data were georeferenced and analyzed using TerraView® Geographic Information System software, version 4.2.2. The georeferenced meshes in shapefile format (.shp) of the municipal boundaries of Pará were obtained from the Brazilian Institute of Geography and Statistics, Horizontal Datum SIRGAS-2000, longlat projection system. Tables were created using Microsoft Excel.

## Results

During the study period, 6,487 hospitalizations for CS were recorded among children under one year of age in Pará, with 23 deaths during hospitalization (0.33%) (Table 1). The early neonatal period (0 to 6 days) accounted for 94% (6,096) of cases, while hospitalizations in the late neonatal period (7 to 28 days) accounted for 3.3% (213) and 2.7% (178) in the postnatal period (29 to 364 days), data not shown.

**Table 1:** Temporal distribution of hospitalizations, lethality and mortality congenital syphilis. Pará. 2009–2018

Year	Live births	Hospitalizations	Death	Lethality rate	Crude hospitalizations rate*	Crude Mortality rate *
2009	143.140	345	3	0,9%	2,4	0,02
2010	140.687	453	2	0,4%	3,2	0,01
2011	141.974	470	3	0,6%	3,3	0,02
2012	137.837	490	2	0,4%	3,6	0,01
2013	139.416	536	4	0,8%	3,8	0,03
2014	143.503	742	2	0,3%	5,2	0,01
2015	143.657	727	1	0,1%	5,1	0,01
2016	137.681	850	2	0,2%	6,2	0,01
2017	138.684	880	1	0,1%	6,3	0,01
2018	140.007	994	3	0,3%	7,1	0,02

* 1,000 live births

Table 2 presents the temporal trend analysis. For the ten (10) years of the study period, it appears that despite the increase in the crude hospitalization rate due to CS, there was a reduction in the lethality rate, but there was variation in the mortality rate due to CS among hospitalized patients. The lethality rate, between 2009 and 2018, showed a decreasing trend (APC: -13.5%; 95% CI: -22.7; -3.3; *p* = 0.01). The crude hospitalization rate for CS, between 2009 and 2018, showed an increasing trend (APC: 11.9%; 95% CI: 9.9; 13.9; *p* < 0.000). The regression analysis demonstrated that there was a change point in the trend with a significant growth of 12.8% until 2016, (95% CI: 8.3; 17.6; *p* = 0.0006). Subsequently, from 2016 to 2018, the annual percentage change was 8.1%, however there was no statistical evidence (95% CI: -14.1; 36.1; *p* = 0.43). In the mortality rate the trend was stable (APC: -2.9%; 95% CI: -12.4;7.7; *p* = 0.56) (Table 2).

**Table 2:** Temporal trend analysis of hospitalizations, lethality and mortality congenital syphilis. Pará. 2009–2018

Variables (indicator)	Year	APC	CI 95%	p value
Lethality rate	2009–2018	-13.5%	22.7; -3.3	0.01
Crude hospitalizations rate*	2009–2018	11.9%	9.9;13.9	< 0.0001
	2009–2016	12.8%	8.3;17.6	0.0006
	2016–2018	8.1%	-14.1; 36.1	0.43
Crude Mortality rate *	2009–2018	-2.9%	-12.4;7.7	0.56

* 1,000 live births. APC: Annual Percentage Change. CI: confidence interval

**Fig. 2:**
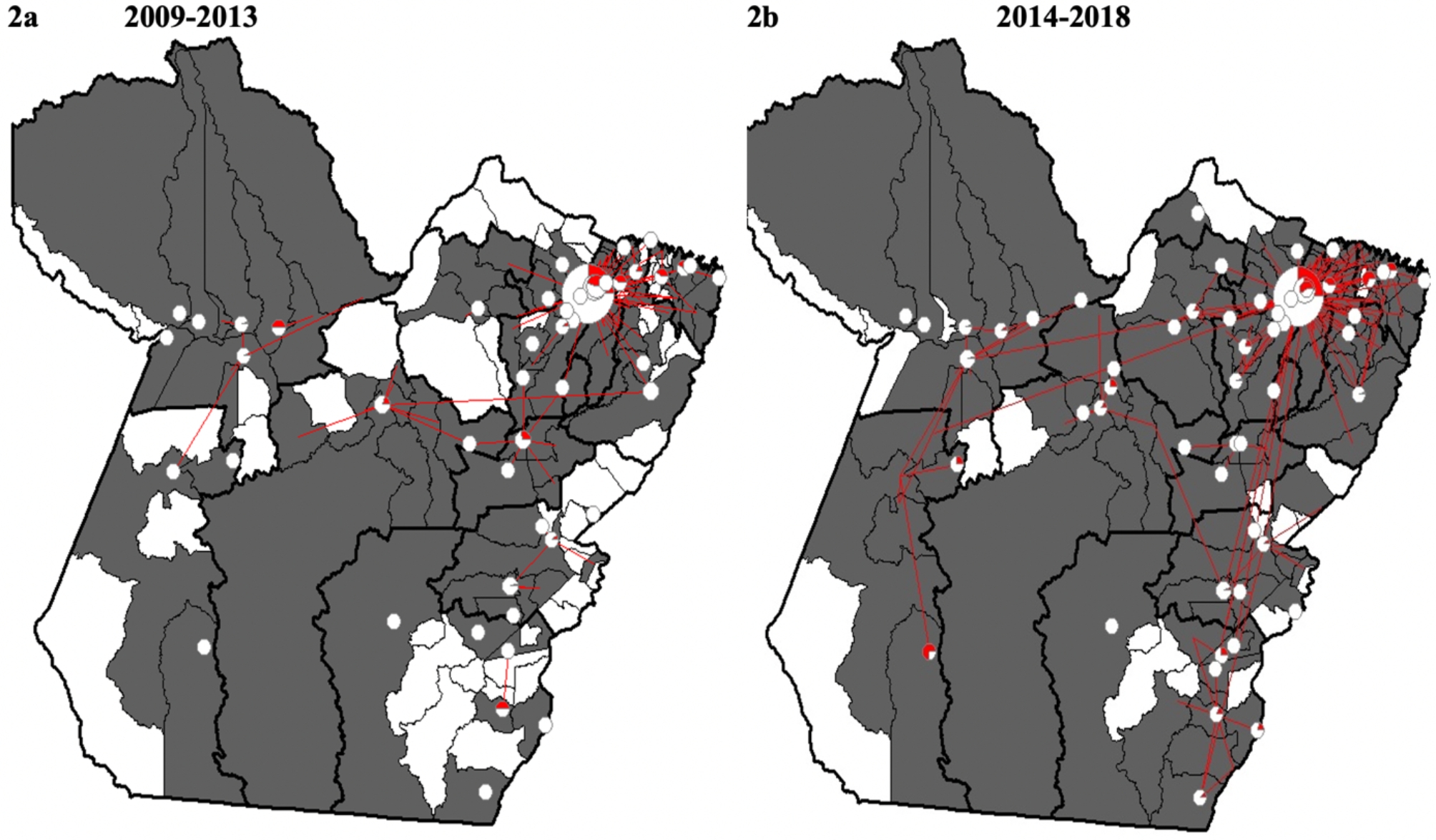
Mapping of local and dominant flows of hospitalizations due to CS. Dark line – health region. Pie charts: red color - proportion of hospitalizations of external origin. White color for admissions at the place of residence. **2a**. 2009–2013 and **2b**. 2014–2018. Circles: local links. Arrows: Dominant external flows. The map was constructed by the authors (elaborate in Tabwin program 4.15)

The analysis of hospitalization care flows made it possible to identify that most hospitalizations due to CS occurred in the municipalities of residence, but 1,378 (21.2%) had to move, with a lethality rate of 0.5% (07/1378). Between 2009 and 2013, 155 hospitalization flows (2,294) were identified, of which 108 were external flows (links between municipalities) which corresponds to 19.4% (446) of hospitalizations. In the following period, there were 234 flows (4,193), of which 181 were external flows (22.2%; 932). The largest flow was from the municipality of Ananindeua to Belém in all periods, both from Metropolitana I (Fig. 2).

Table 3 presents the capacity of health regions to admit their residents or admit residents to another health region (reference), in addition to the capacity to admit residents from other health regions. The analysis shows that the Metropolitan I health region did not need to refer its residents to hospitals in other health regions. However, there was a high number of hospitalizations of residents from other regions. Meanwhile, in the period studied, Metropolitana II managed to admit only one resident in the region itself. All residents who needed hospitalization were referred to other regions. The Baixo Amazonas, Araguaia and Carajás health regions managed to cover all hospitalizations of residents of the region in the period 2009–2013. In the second period of 2014–2018, there was an increase in the number of residents who needed hospitalization for CS, but the number of referrals for hospitalization in other regions was low.

**Table 3:** Flow of hospitalizations for CS in children under one year of age, according to the capacity of health regions to hospitalize their residents and those of other regions. Pará. 2009 to 2013 and 2014 to 2018

Health region	Total hospitalizations of residents of the region health		Total of hospitalizations in the health region		Hospitalization of residents in another health region		Hospitalization of residents from other health regions		Balance*	
	2009–2013	2014–2018	2009–2013	2014–2018	2009–2013	2014–2018	2009–2013	2014–2018	2009–2013	2014–2018
Macroregion I										
Metropolitana I	1.142	2.303	1.284	2.729	0	0	142	426	142	426
Marajó I	19	36	10	7	9	29	0	0	-9	-29
Marajó II	76	111	73	105	3	6	0	0	-3	-6
Tocantins	212	339	181	262	31	77	0	0	-31	-77
Macroregion II										
Metropolitana II	49	135	1	0	48	135	0	0	-48	-135
Metropolitana III	156	232	121	96	38	136	3	0	-35	-136
Rio Caetés	77	118	67	93	10	30	0	5	-10	-25
Macroregion III										
Baixo Amazonas	157	325	159	322	0	5	2	2	2	-3
Tapajós	16	12	14	9	2	3	0	0	-2	-3
Xingú	102	79	100	77	2	2	0	0	-2	-2
Macroregion IV										
Araguaia	9	73	9	70	0	3	0	0	0	-3
Carajás	159	297	159	293	0	6	0	2	0	-4
Lago de Tucuruí	120	133	116	130	6	4	2	1	-4	-3

*Balance = Hospitalizations of residents from other health regions minus Hospitalizations of residents in another health region

For analysis of network mapping, hierarchy and classification of flows, networks that attended 10 or more hospitalizations per CS were considered. Eight main assistance networks were found, with Ananindeua formed in the second period. In Macroregion I, Metropolitan Health Region I had all types of flows and the largest number of municipalities forming care networks, Belém, Marituba and Ananindeua, in addition to serving the largest number of health regions and almost all the demand of Metropolitana II, belonging to the other macroregion. Among the other health regions, only the Tucuruí care network hospitalized residents from other macroregions (Table 4).

**Table 4:** Hospitalization flow for care networks, according to the type of flow. Pará. 2009 to 2018

Assistance network and region of residence	Type of hospitalization flow					
	The flow follows the network structure *		Flow occurs between different networks **		Flow occurs between different networks and connects the same hierarchical level ***	
	2009–2013	2014–2018	2009–2013	2014–2018	2009–2013	2014–2018
Belém						
Metropolitana I	96	0	6	19	14	179
Marajó I	9	26	0	0	0	0
Marajó II	3	0	0	3	0	1
Tocantins	29	72	0	0	0	1
Metropolitana II	15	63	9	14	0	0
Metropolitana III	25	116	0	0	10	0
Rio Caetés	7	22	0	4	1	4
Baixo Amazonas, Tapajós, Xingú	0	1	0	0	1	5
Lago Tucuruí e Carajás	6	1	0	0	0	4
Ananindeua						
Metropolitana I	0	25	0	1	0	63
Marajó I	0	0	0	0	0	0
Marajó II	0	0	0	1	0	0
Tocantins	0	0	0	3	0	0
Metropolitana II	0	0	0	19	0	0
Metropolitana III	0	0	0	7	0	0
Marituba						
Metropolitana I	31	8	14	18	7	37
Metropolitana II	17	20	4	18	0	0
Metropolitana III	0	0	2	6	0	0
Carajás	0	0	0	0	0	1
Breves						
Marajó II	1	12	0	0	0	0
Bragança						
Rio Caetés	22	29	0	2	0	0
Metropolitana II e III	0	0	0	3	0	0
Altamira						
Xingú	15	8	1	0	0	0
Parauapebas						
Carajás	2	11	0	1	2	1
Tucuruí						
Tocantins e Xingú	2	0	0	0	0	0
Lago Tucuruí	28	10	0	0	0	0

HDA*: Hierarchical direct ascending (The flow follows the network structure and the link goes to a node at a higher hierarchical level); ATN**: Ascending transversal between nets (When the flow occurs between different networks, going to a node at a higher hierarchical level); HTN***: Horizontal transversal between nets (When the flow occurs between different networks and connects nodes at the same hierarchical level)

When considering the distances traveled to assess access in both periods, the greatest distances traveled by residents of Macroregion III (Xingú and Tapajós) to hospitalization in Belém, also presenting the greatest distances to hospitalization in the same health region, 258 km, a an HDA flow from Almeirim to Santarém (Baixo Amazonas). The smallest distances were covered by residents of Metropolitana I (Table 5).

**Table 5:** Distance traveled to be hospitalized for CS, according to the health region. Pará. 2009 to 2013 and 2014 to 2018

Health region	Shortest distance (Km)*		Longest distance (Km)**		Average distance (Km)***	
	2009–2013	2014–2018	2009–2013	2014–2018	2009–2013	2014–2018
Macroregion I						
Metropolitana I	4	4	33	33	17	17
Marajó I	43	43	119	119	78	85
Marajó II	29	29	170	223	116	111
Tocantins	16	16	169	198	78	80
Macroregion II						
Metropolitana II	20	20	113	154	58	66
Metropolitana III	20	19	212	278	91	112
Rio Caetés	14	14	218	262	123	122
Macroregion III						
Baixo Amazonas	34	28	258	700	92	172
Tapajós	0	48	247	796	247	303
Xingú	38	33	457	322	150	116
Macroregion IV						
Araguaia	0	21	103	673	103	205
Carajás	32	33	116	583	74	187
Lago de Tucuruí	11	11	172	172	88	77

Km: kilometer. * Shorter distance: shorter distance (straight line between place of residence and place of hospitalization) of hospitalizations of residents of the health region. **Greater distance: greater displacement (straight line between place of residence and place of hospitalization) of hospitalizations of residents of the health region. ***Average distance: average distance of hospitalizations (straight line between place of residence and place of hospitalization)

## Discussion

In the present study, different analysis methods were used to investigate the magnitude of hospitalizations for CS in Pará in children under one year of age from 2009 to 2018. The use of the disaggregated indicator, with only data on hospitalizations for CS, made it possible to evaluate the resolution of PHC for SC and the use of hospital care in Pará. The results of the crude hospitalization rate, mortality rate during hospitalization and lethality rate due to SC demonstrate different trends in the period studied. These result indicators make it possible to monitor the performance of national health systems, PHC and the coordination of care between different levels of health care [7, 14].

As found in a previous study carried out in Brazil, between 2000 and 2015, the result of the temporal trend analysis of the CS hospitalization rate showed an increasing trend [6]. This trend in CS hospitalization rates indicates that there are still failures in PHC, specifically in prenatal care, even after the implementation of the “stork network”. In this country, the increase in syphilis cases was associated with greater availability of rapid tests for detection in the primary care network, however, with less availability of condoms and penicillin and teams that do not administer the antibiotic [22]. In addition to the organization of health services, global situations can have local impacts, even if temporary, on syphilis control strategies, such as the shortage of global penicillin stocks that occurred between 2014 and 2016 [23].

It is important to emphasize that the improvement of a complex outcome indicator such as hospitalizations for CS depends on several factors that include structural interventions in public health policies to eliminate congenital syphilis implemented at the national level [1, 2, 24]; in the organization of health services to serve pregnant women and their partners [22]; interventions on the most exposed populations, such as those from countries with low socioeconomic indicators [24] who start prenatal care late [25], such as inadequate treatment [3, 8]; marital status single/divorced [26].

In addition to Brazil, other countries in the Americas, such as Chile, Paraguay, Dominican Republic, Peru, Argentina, Trinidad and Tobago, have also prioritized structural interventions in health policies, such as the provision of rapid tests and penicillin in PHC; surveillance of sexual partners, monitoring the treatment of pregnant women with syphilis and investigation of CS cases; and improvements in health information systems [2]. However, it is clear that rate of congenital syphilis in Latin America and the Caribbean are still high [1, 24]. In England, which has a national health system with universal access, preventable hospitalizations through PHC and mortality showed small reductions even after a series of national government measures were implemented to improve access and quality of this level of care [14].

The reduction in the CS mortality rate with a decreasing trend shows the best results in tertiary care, since the increase in hospitalizations did not result in an increase in the number of deaths, even among those who had to move to other municipalities. These results could be more critical when considering the large percentage of children hospitalized for CS in the early neonatal period, who are at greater risk of dying when access to health services is difficult [6, 9, 11]. Studies show that investments by the “stork network” in Brazil enabled the qualification of obstetric and neonatal beds, expansion of obstetric residency programs, changes in the care model, access to appropriate technology for childbirth and reduction of practices considered harmful [27, 28].

However, in PHC the implementation of the “stork network” had no impact on financing that would qualify maternal and child health. During the study period, the basic care floor was in force based on population criteria, per capita, and other incentives. In 2019, this type of financing was replaced by weighted capitation, payment for performance, incentives for strategic actions and provision of health professionals. In this new modality, in payment for performance there are indicators for prenatal care and one specific for syphilis, which evaluates the performance of tests for syphilis and HIV among pregnant women [29, 30].

The population coverage of PHCs implies access to primary care and other levels of health care, but this integration of PHCs into the care network continues to be a challenge [31, 32], even after the implementation of the “stork network” [4] and the regulation system that minimized systemic problems of access to care for PHC demands for hospitalization, interconnecting the different points of care, even if they are in different territories [33], being important in the organization of the Health Care Network and for flow assistance [34, 35].

Next, spatial analysis techniques were applied to identify whether there were changes provided by the “stork network” on the travel route and travel distance of newborns who needed to be hospitalized due to CS. In the literature, no study was found on hospital admission for CS using these techniques. Several studies have been arried out on the flow of care for oncology hospitalizations, chemotherapy and radiotherapy [35–38], heart surgery [39], childbirth [40], and organ transplantation services [41].

The results demonstrate that after the implementation of the “stork network” there was an increase in the capacity of regions and macroregions to admit residents to their own territories. Previous studies have highlighted the dynamics of regionalization based on hospitalization flows between municipalities and health regions [34, 35, 37], with the evolution of the hospitalization network for causes of hospitalization, such as heart surgery and oncology [36, 39].

However, in the present study, two large care gaps were highlighted in Metropolitan health regions II and III, belonging to macroregion II. The hospitalizations of residents of these regions were carried out by the assistance networks of Belém (capital) and Marituba, both of which are part of Metropolitana I. This is the region with the greatest sufficiency to admit its residents, with assistance networks having mainly hierarchical flows, functioning in accordance with the patient referral mechanisms in place. This type of flow was found in the South and Southeast regions in chemotherapy treatment, whose care network is more organized than the North [37].

The care network in Belém, Ananindeua and Marituba has the largest number of obstetric and neonatal beds in the state and the main regulatory centers [42]. Previous studies have also identified a greater flow of hospitalizations toward centers and centers that have qualified hospitals in terms of physical structure, credibility of the population, support infrastructure and good clinical results, mainly in the Southeast region of Brazil [35, 36]. In Bahia, an expansion of the hospital network outside the capital was observed. In 2010, the municipality of Vitória da Conquista had records of patients coming from just 8 different municipalities, and as of 2014, hospitalizations of patients from 5 different macroregions were recorded [39].

The greater concentration of services in urban areas highlights inequalities in the distribution of the health service network and population access [10, 11, 33, 36]. However, this concentration, which imposes a greater flow of hospitalizations toward the host cities [36], must be evaluated in terms of overload and reduction in quality, which can be measured by indicators such as fatality rate and hospital mortality [11]. Municipalities and health regions must be preceded by analyzing existing agreements and complying with access regulation protocols to guarantee care and avoid pilgrimages [11, 20].

Residents of macroregions III and IV had the greatest distances traveled to access hospital care. The municipalities located in these macros have large territorial extensions [19]. This characteristic may be related to average distances traveled greater than 100 km in six reference municipalities in Rio Grande do Sul [38]. There is a need to prioritize access over economies of scale in the programming and agreement of health actions and services [4, 43]. The basis of economies of scale was applied to the expansion of the hospital network from the perspective of regionalized and hierarchical health care networks. It involves a health region offering a certain service to several other regions and everyone benefits from lower installation and maintenance costs [43].

Difficulties in PHC access to referral units and hospitals were found in the upper east region of Ghana. Among 100 PHC clinics studied, only 15% were located less than 10 km from their nearest referral facility. The majority (66%) of PHC clinics were located 15 to 40 km away from the nearest referral hospital/medical laboratory [44]. In this country, for radiotherapy this displacement increases, with 70% living 200 km from a facility that offers radiotherapy [45].

The lack of organization of regionalized care networks and flows means that the population in the northern Brazil faces barriers in accessing health services, traveling more than 1,000 km to receive different types of cancer treatment [36]. The analysis of hospitalization flows for breast cancer, chemotherapy and radiotherapy also identified that the highest median distances traveled are in the states in the Brazilian Amazon regions. In Amapá, Roraima and Amazonas, displacements are greater than 500 km and in the State of Pará the displacement can reach 1,000 km. However, the states in the Southeast and South regions may be less than 250 km [35].

The present study demonstrates that despite the long distances, the number of patients who had to travel to be admitted to other regions was lower than those who were admitted to the health region itself. These results differ from those found in a previous study; in the care network for chronic conditions related to cancer, more than half of the patients had to travel from their home municipalities to receive treatment and Brazilian regional disparities in accessibility persist over time [36]. Geographic and infrastructure differences are also partially responsible for disparities in the provision of transplant services in the Brazilian Amazon region. The South and Southeast have the highest service coverage [41].

The study revealed that despite the increasing trend in hospitalizations for CS in the studied period, there was a decreasing trend in the mortality rate and a higher number of hospitalizations in the health regions/macroregion of residence. This demonstrates the impact of the lack of timely diagnosis and treatment of undiagnosed prenatal syphilis. However, tertiary care was able to absorb demand without increasing deaths, even in adverse geographic conditions for population displacement.

As in other studies, the analysis of flows and mapping of care networks proved to be a health planning and management tool for the Health Care Network. Using these techniques, it is possible to analyze the potential for optimizing geographic access in the network of health care based on programming and simulation and scenarios compared to the current configuration or for the installation of new services [36, 38]. The analysis of network flows makes it possible to evaluate intermunicipal networks that have already been installed [34, 35].

This study has limitations in the use of secondary data to indirectly calculate the distances traveled for commuting, as well as the characteristics of the region that limit the generalization of the results to other territories.

## Conclusion

The increase in the rate of hospitalizations with a tendency to increase demonstrates the impact that syphilis still causes in Brazil, not being resolved even after national government interventions. The results demonstrate a heterogeneous pattern in the distribution of municipalities of residence of people who were hospitalized and in the organization of the Health Care Network within the health regions and macroregions of the state. It is noted that although the state has a structured care network for children under one year of age who require hospitalization due to CS, care gaps were still observed in metropolitan regions II and III, and long distances traveled were related to the large territorial extension of some municipalities in the macroregions III and IV.

The results of the study highlight the need for managers to qualify primary health care in physical infrastructure and expand population coverage by family health teams or in riverside/river models and evaluate maternal and child indicators related to CS. Furthermore, in hospital care, the expansion of beds or services in places that are difficult to access due to travel. In the context of policies, specific financing modalities that take into account the peculiarities of the Brazilian Amazon are essential, as well as the expansion of actions for the region, as occurs with the modalities of family health teams.

## Data Availability

The datasets generated and/or analysed during the current study are available in the DATASUS repository, http://sihd.datasus.gov.br/principal/index.php and TabNet repository, http://tabnet.datasus.gov.br/cgi/deftohtm.exe?sinasc/cnv/nvpa.def. Platform with access provided by Ministry of Health. The datasets used during this current study are also available from the corresponding author on reasonable request.

## Abbreviations

CS: Congenital syphilis
GS: gestational syphilis
PHC: Primary Health Care
SUS: (In portuguese) Unified Health System
HR: health regions
APC: Annual Percentage Change
CI: confidence interval
HDA: hierarchical direct ascending
HTN: horizontal transversal between networks
ATN: Ascending transversals between networks
